# Supplementary material for: New Mitogenome Features of Philopotamidae (Insecta: Trichoptera) with Two New Species of Gunungiella
Source: Insects. 2022 Nov 29;13(12):1101. doi: 10.3390/insects13121101 (PMC9784110; doi:10.3390/insects13121101)
Supplement: Supplementary file 1 [file insects-13-01101-s001.zip › Supplementary Materials.pdf]

Table S1. Detailed taxonomic resources used in present study.

Subfamily	Subfamily	Genus	Species	GenBank accession number
Outgroup				
Philopotamidae	Chimarrinae	*Chimarra*	*Chimarra paramonorum* Hu, Sun &Wang, 2018*	OL678008
			Chimarra sadayu Malicky, 1993	OP351513
	Philopotaminae	*Dolophilodes*	*Dolophilodes bellatula* Sun & Malicky, 2002*	OL678011
		Gunungiella	*Gunungiella acanthoclada* Sun, 2007	OP351514
			Gunungiella flabellata sp. nov	OP351515
			Gunungiella wangi sp. nov	OP351516
		Kisaura	*Kisaura zhejiangensis* Hu, 2019*	OL678025
			Kisaura adamickai Sun & Malicky, 2002	OP351518
		Wormaldia	*Wormaldia unispina* Sun, 1998*	OL678056
			Wormaldia spinosa Ross, 1956	OP351517
Ingroup				
Hydropsychidae	Arctopsychinae	*Arctopsyche*	*Arctopsyche spinescens* Gui & Yang, 2001*	ON859124
			Arctopsyche sp.*	ON859111
			Parapsyche elsis Milne, 1936*	ON859123
	Diplectroninae	*Diplectrona*	*Diplectrona albofasciata* (Ulmer, 1913)	ON859114
			Diplectrona hexapetala Sun, 2012*	OL678009
	Hydropsychinae	*Hydromanicus*	*Hydromanicus huapingensis* (Li & Tian, 1990)*	ON859115
			Hydromanicus melli (Ulmer, 1926)*	OL678021
		Hydropsyche	*Hydropsyche cerva* (Li & Tian, 1990)*	ON859117
			Hydropsyche formosana Ulmer, 1911*	ON859118
			Hydropsyche fryeri Ulmer, 1915*	NC_060325
			Hydropsyche fukienensis Schmid, 1965*	OL678022
			Hydropsyche rhomboana Martynov, 1909*	ON859120

			Hydropsyche trifora (Li & Tian, 1990)*	ON859122
Stenopsychidae	Stenopsychinae	*Stenopsyche*	*Stenopsyche angustata* Martynov, 1930*	NC_051529
			Stenopsyche navasi Ulmer, 1926*	OL678052
			Stenopsyche tienmushanensis Hwang, 1957*	MW201980

* Published mitogenomes of Trichoptera.

Table S2. Nucleotide composition of mitogenomes of six philopotamid species.

	Species	Whole genome	PCGs	FCP	SCP	TCP	tRNAs	l-rRNA	s-rRNA	CR
Length(bp)	*Chimarra sadayu*	16602	11096	3701	3699	3696	1448	1255	802	1994
	Gunungiella flabellata	15338	11106	3704	3700	3702	1479	1258	763	641
	Gunungiella acanthoclada	15657	11108	3705	3703	3700	1405	1258	764	980
	Gunungiella wangi	15333	11104	3703	3701	3700	1489	1258	757	652
	Kisaura adamickai	15949	11112	3706	3704	3702	1458	1360	765	1221
	Wormaldia spinosa	15181	11132	3712	3711	3709	1456	1321	748	543
A+T (%)	*Chimarra sadayu*	77.75	75.5	74.05	72.32	84.75	83.08	81.51	84.04	81.34
	Gunungiella flabellata	73.63	69.63	71.13	71.62	76.95	82.69	80.68	85.71	93.45
	Gunungiella acanthoclada	76.11	72.17	70.77	71.50	68.72	82.78	82.51	86.52	93.37
	Gunungiella wangi	75.51	72.01	72.50	71.70	75.26	83.68	81.72	86.39	91.26
	Kisaura adamickai	81.95	79.92	77.54	74.16	90.88	83.81	85.51	87.84	90.01
	Wormaldia spinosa	77.96	75.41	73.41	72.55	84.39	83.17	83.57	85.43	93.19
AT-Skew	*chimarra sadayu*	0.01	-0.13	0.05	-0.39	-0.06	0.02	-0.02	0.05	-0.04
	Gunungiella flabellata	0.06	-0.14	0.03	-0.40	0.02	0.00	0.00	0.05	0.06
	Gunungiella acanthoclada	0.04	-0.14	0.03	-0.39	0.01	0.01	0.00	0.04	0.01
	Gunungiella wangi	0.03	-0.15	0.01	-0.40	-0.02	-0.01	0.01	0.05	0.03

	Kisaura adamickai	-0.02	-0.12	0.02	-0.37	-0.03	0.00	0.01	0.04	-0.03
	Wormaldia spinosa	0.00	-0.13	0.03	-0.38	-0.04	0.02	0.01	0.05	-0.05
GC-Skew	*chimarra sadayu*	-0.26	0.00	-0.07	-0.04	0.24	0.13	0.38	0.39	0.24
	Gunungiella flabellata	-0.46	-0.09	0.00	0.17	0.40	0.05	0.50	0.43	-0.24
	Gunungiella acanthoclada	-0.43	-0.09	0.02	0.17	0.45	0.07	0.48	0.38	0.02
	Gunungiella wangi	-0.42	-0.08	-0.03	0.16	0.41	0.14	0.49	0.39	-0.21
	Kisaura adamickai	-0.25	-0.01	-0.09	0.09	0.35	0.14	0.31	0.31	-0.11
	Wormaldia spinosa	-0.36	-0.07	0.02	0.12	0.39	0.09	0.41	0.32	-0.22

Abbreviations: FCP: First codon position; SCP: Second codon position TCP: Third codon position.

Table S3. PCR primers used to sequence *mtCOI* genes of Trichoptera in this study.

Primer	Sequence
LCO1490	GGTCAACAAATCATAAAGATATTGG
HCO2198	TAAACTTCAGGGTGACCAAAAAATCA

Table S4. The best model for each partition of the three Matrixs.

Matrixs	Partition names	Best model
PCG12	*ATP6*, *COX2*, *COX3*, *CYTB*	GTR+F+I+G4
	ATP8, *ND2*, *ND3*, *ND6*	GTR+F+I+G4
	COX1	TIM2+F+I+G4:
	ND1, *ND4*, *ND4L*, *ND5*,	GTR+F+I+G4
PCG12R	16S, 12S	GTR+F+I+G4
	ATP6, *COX2*, *COX3*, *CYTB*	GTR+F+I+G4

	ATP8, *ND2*, *ND3*, *ND6*	GTR+F+I+G4
	COX1	TIM2+F+I+G4:
	ND1, *ND4*, *ND4L*, *ND5*,	GTR+F+I+G4
	ATP6, *ATP8*, *COX2*, *COX3*, *CYTB*, *ND2*, *ND3*, *ND6*	mtZOA+F+I+G4:
PCGFAA	*COX1*	mtART+I+G4:
	ND1, *ND4*, *ND4L*, *ND5*	mtMet+F+R4:

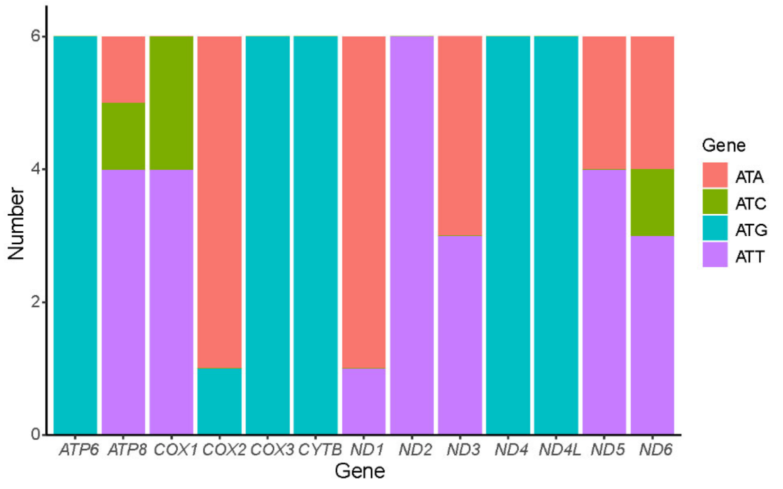

Figure S1. Start codons of PCGs among Trichoptera mitogenomes.

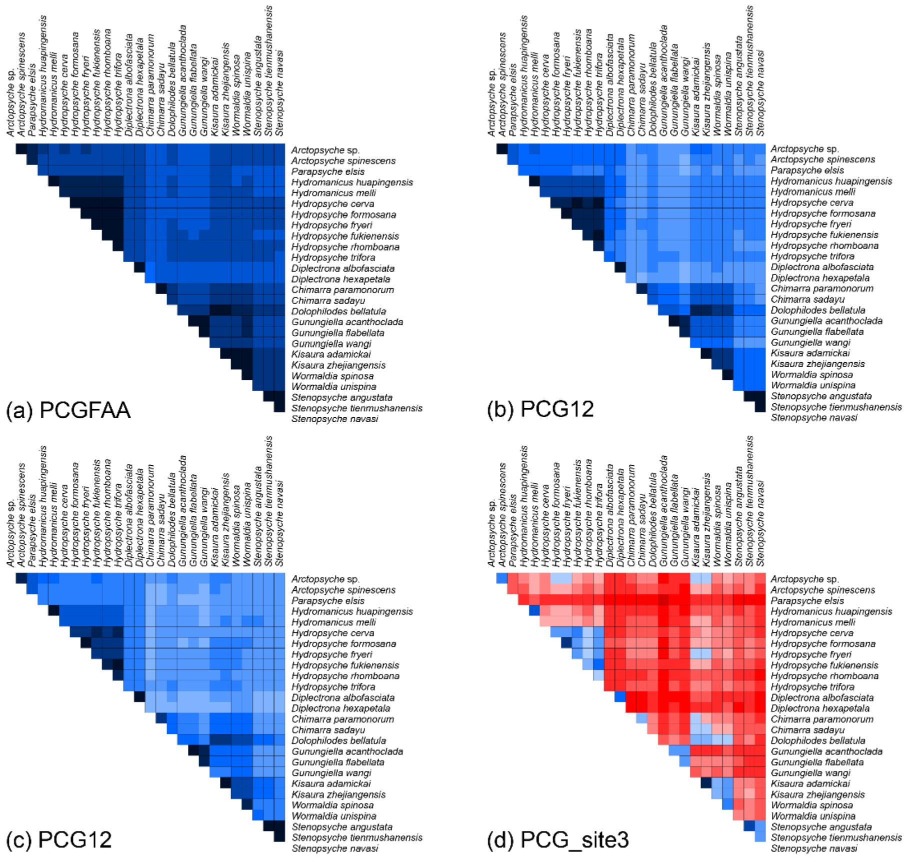

Figure S2. Heterogeneity of sequence composition for different datasets. The pairwise Aliscore values are represented by colored. The scores range from −1 showing full random similarity (dark blue), to +1 showing non-random similarity (shiny red). (a) PCGFAA (b) PCG12R; (c) PCG12; (d) PCGs_site3.
